# Physical, Mechanical, and Durability Performance of Olive Pomace Ash in Eco-Friendly Mortars

**DOI:** 10.3390/ma18112667

**Published:** 2025-06-05

**Authors:** Besma Belaidi, Abderraouf Messai, Cherif Belebchouche, Mourad Boutlikht, Kamel Hebbache, Abdellah Douadi, Laura Moretti

**Affiliations:** 1 Materials and Durability of Constructions Laboratory, Department of Civil Engineering, Faculty of Sciences of Technology, University of Constantine 1 Frères Mentouri, Constantine 25000, Algeria; besma.belaidi@umc.edu.dz (B.B.); cherif.belebchouche@univ-setif.dz (C.B.); 2 Civil Engineering Research Laboratory of Sétif (LRGCS), Department of Civil Engineering, Setif 1 University-Ferhat Abbas, Sétif 19000, Algeria; abderraouf.messai@univ-setif.dz (A.M.); mouradboutlikht@gmail.com (M.B.); hebbache.kamel@univ-setif.dz (K.H.); abdellah.douadi@univ-setif.dz (A.D.); 3 Emergent Materials Research Unit (EMRU), Setif 1 University-Ferhat Abbas, Sétif 19000, Algeria; 4 Department of Civil, Building and Environmental Engineering, Sapienza University of Rome, Via Eudossiana 18, 00184 Rome, Italy

**Keywords:** secondary raw materials, mortar, olive, pomace ash, biomass ash, cement substitute

## Abstract

The cement industry is a major contributor to global CO_2_ emissions, driving the research for sustainable alternatives. Olive biomass ash (OBA), a byproduct from burning all types of biomass from the olive tree, has emerged as a potential supplementary cementitious material (SCM). This study investigates the effects of incorporating olive pomace ash (OPA) as a partial cement substitute (0% to 50% by weight) on mortar properties over extended curing periods. Workability, compressive and flexural strengths, water absorption, and freeze–thaw resistance were evaluated. Up to 20% OPA replacement improved workability while maintaining acceptable strength and durability. Beyond this level, mechanical properties and frost resistance decreased significantly. Correlation analyses revealed strong relationships between flow time and wet bulk density (R^2^ = 0.93), an exponential relationship between 28-day compressive strength and water absorption (R^2^ = 0.87), and linear correlations between pre- and post-freeze–thaw mechanical properties (R^2^ ≥ 0.99 for both compressive and flexural strengths). The results demonstrate that optimal OPA incorporation enhances mortar performance without compromising structural integrity and provides a viable strategy for valorizing agricultural waste.

## 1. Introduction

Cement production exceeds 4 billion tons annually, contributing approximately 8% to global CO_2_ emissions [1] due to high-temperature processing and limestone calcination [2]. In response to increasing environmental concerns [3], greener binders based on supplementary cementitious materials (SCMs) have become a pressing need. Industrial byproducts, such as fly ash, silica fume, and slag, have been extensively studied; however, alternative biomass-derived ashes remain underexplored [4].

Biomass ashes demonstrate pozzolanic actions ideal for optimizing concrete attributes and sustainability [5]. Olive biomass ash represents a promising SCM among biomass ashes, given the vast quantities generated by the global olive oil industry, particularly in Mediterranean regions [6]. Olive pomace, a residual biomass from olive oil extraction, when thermally processed, yields an ash rich in silica and other pozzolanic compounds. Biomass ash SCMs can curtail the cement factory and embodied carbon footprint of concrete while also furnishing a waste valorization outlet for biomass ashes otherwise destined for landfills [7]. Although preliminary studies have indicated potential benefits in enhancing durability and mechanical properties, knowledge gaps persist regarding long-term performance and durability under aggressive environmental conditions [8]. Therefore, systematic research tailored to each spectrum of biomass residues is imperative to harness their application potential as eco-friendly SCMs.

Olive waste ash (OWA) shines as a remarkably high-potential yet understudied biomass ash SCM, courtesy of the expansive global olive oil business, which churns out millions of tons of this agricultural waste annually [9]. The annual world olive oil output surpasses 3 million metric tons, with premier producers around the Mediterranean basin encompassing European countries, like Spain, Italy, and Greece [10]. The scale of global olive farming necessitates substantial olive waste generation, with over 10 million hectares of olive groves worldwide [11]. Consequently, the countries flanking the Mediterranean Sea produce millions of tons of OWA yearly [12]. Historically, these leftover materials were disposed of through practices like landfills or open incineration, leading to environmental pollution. Nevertheless, the lignocellulosic characteristics of olive stones and their notable silica content have recently drawn attention due to their potential applications in the construction and development of building materials [4,13].

Over the last decade, numerous investigations have examined the integration of OWA into cementitious binders and mortar/concrete systems. Al-Akhras and Abdulwahid [14] examined mortar compositions that featured the addition of OWA, replacing portions of sand and cement. The substitution of sand with 5–20% OWA boosted the 28-day compressive strength (CS28), escalating from 26.5 to 38.2 MPa. However, the cement replacement reduced the strength because of the dilution of cementitious compounds. Incorporating OWA enhanced durability against alkali–silica reactions, with improvements observed up to a 20% addition [15], and resistance to high temperatures [16].

Cuenca et al. [17] reported that including 10–15% OWA upheld the compressive strength of self-compacting concretes similar to control compositions. Microstructure analysis disclosed refinements in the distribution of pore sizes and disconnection between pores. Eliche-Quesada and Leite-Costa [18] employed OWA to substitute natural sand in fired clay bricks, leading to better thermal insulation and mechanical performance. Beltran et al. [19] determined that introducing OWA into masonry mortars increased porosity and water absorption, albeit with a slight reduction in mechanical strength. They advised regulating workability and strength by adjusting the fineness and texture of OWA particles. Font et al. [20] synthesized binders consisting of 100% OWA–slag, revealing notable early strength development and resistance against acid attacks. Payá et al. [21] modified the reactivity of OWA through thermal and chemical treatments to optimize its performance as a supplementary cementitious material in both fresh and hardened states.

Collectively, the prior research underscores the promising potential of OWA as a sustainable substitute for cement at moderate proportions, effectively enhancing the attributes and ecological profile of cementitious systems. However, there are still significant knowledge gaps, particularly regarding the long-term properties and durability performance of OWA-modified concretes. Most studies have been limited to short-term analysis without systematically evaluating the influence of OWA across extended curing ages. Moreover, in-depth investigations correlating the microstructure and durability indicators are lacking.

This study investigates the properties of mortars incorporating olive pomace ash (OPA) by varying curing periods and replacement ratios. Olive pomace ash (OPA) is the byproduct after olive oil extraction, typically a mix of olive skins, pulp, and crushed pits. It is added at replacement levels of 0%, 10%, 20%, 30%, 40%, and 50% by weight of cement. The effect of different OPA ratios on the compressive and flexural strength development at 7, 28, and 90 days is analyzed. The mortars’ water absorption, wet bulk density, flow time, and freeze–thaw resistance are evaluated to determine the durability performance. Correlation analyses further elucidate the relationships among key parameters. The findings aim to advance the sustainable use of OPA in eco-friendly construction materials.

## 2. Materials and Methods

### 2.1. Raw Materials

The binder used in this study was an Ordinary Portland Cement (OPC) conforming to CEM I 42.5 R, sourced from the Biskria plant in Algeria and compliant with the NA 442-2013 standard [22]. Its mineralogical composition, derived from Bogue calculations [23], included tricalcium silicate (C3S) at 65.15% and dicalcium silicate (C2S) at 8.45%, with the remainder comprising calcium aluminate and aluminoferrite phases. The OPC had a specific surface area of 3510 cm^2^/g and a specific gravity of 3.09 g/cm^3^. The X-ray Fluorescence revealed a composition rich in CaO (63.20%) and SiO_2_ (19.39%), consistent with typical Portland clinker chemistry (Table 1).

The olive pomace ash (OPA) was collected from an olive oil extraction facility in Zardaza, Algeria. The residual olive waste was incinerated and ground to produce a fine powder. The OPA had a specific surface area of 4811 cm^2^/g and a specific gravity of 2.18 g/cm^3^. Chemical analysis revealed a high content of CO_2_ (53.2%), along with significant amounts of lime (10.1%) and silica (11.50%), and smaller fractions of iron, aluminum, and magnesium oxides, as presented in Table 2.

**Figure 1:**
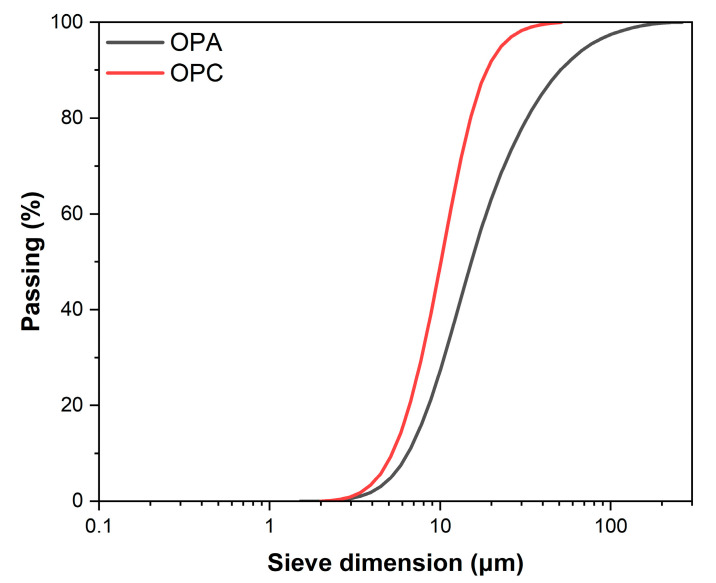
Particle size distribution of OPC and OPA.

Figure 1 reveals key differences between the particle size distribution of OPC and OPA. The OPC particles range from 6.45 μm at the 10th percentile to 50.69 μm at the 90th percentile, with a median size of 23.9 μm. In contrast, the OPA exhibits a finer distribution, spanning 5.23 μm to 18.83 μm for the 10th and 90th percentiles, respectively, and a median of 11.36 μm. The extensive grinding process is responsible for the OPA’s finer particle size. The OPC has a more spread-out distribution, extending to larger sizes, typical for cement manufactured through clinker grinding.

The fine aggregate used was standard siliceous sand, compliant with EN 196-1 [24]. The sand was clean, naturally sourced, and displayed a sub-rounded morphology, suitable for mortar production.

### 2.2. Mix Formulations

Six mortar mixtures were prepared with varying proportions of OPC and OPA, as per EN 196-1 specifications [24]. Table 3 presents the mix proportioning by weight. The control mixture (MC) contained 100% OPC, while the other mixtures were designed with partial replacement of OPC by 10% to 50% OPA in increments of 10%, added by weight of cement. The binder (OPC + OPA)-to-fine-aggregate ratio was maintained at 1:3 for all mixtures. Water was added to achieve a constant water-to-binder ratio (W/B) of 0.5 by weight.

**Figure 2:**
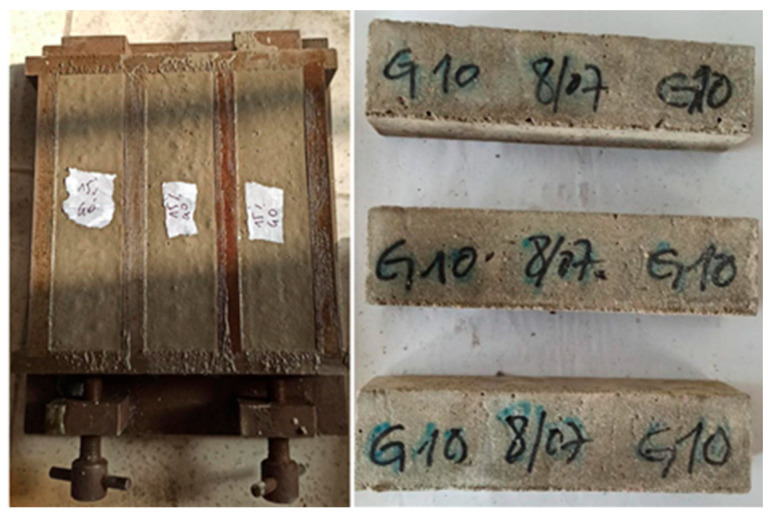
Prismatic specimens.

Mortars were cast in three layers into 40 × 40 × 160 mm^3^ prismatic molds, with each layer compacted to minimize air entrapment. After 24 h of curing under ambient conditions, the specimens were demolded and submerged in water at 23 °C until the testing day (Figure 2).

### 2.3. Experimental Testing Procedure

The Flow Time (FT) Test measures the time a fixed volume of mortar takes to flow and reach a specified mark under standard vibration according to ASTM C1437 [25]. A conical mold was filled with fresh mortar in two layers, each tamped 10 times. Upon mold removal, the spread time to a 100 mm diameter on a vibrating table was recorded. The time between mold lift-off and when the mortar front touched the 100 mm mark was recorded as the flow time.

The bulk density of fresh mortar was determined as per EN 1015-6 [26]. A known volume of mortar was compacted into a pre-weighed 1 L container. The mass of the container plus the wet mortar was recorded. The net mass of the mortar was divided by the volume to calculate the wet bulk density in kg/m^3^. A lower density indicated improved particle packing and workability. The test was conducted at the same flow table consistency used for the molding specimens.

According to [24], a 3000 kN capacity universal testing machine was employed to perform compressive and flexural tests at 7 days, 28 days, and 90 days, using prismatic specimens (40 × 40 × 160 mm^3^). The ultimate flexural and compressive strength was ascertained by averaging the strength values obtained from three samples per mix.

The water absorption (WA) test was conducted according to EN 13057 [27]. Three identical specimens were selected for each mixture. These specimens underwent drying at 105 ± 5 °C until a consistent mass was attained, following EN 13057 requirements [27]. Subsequently, the dried specimens were immersed in water for 24 h to achieve full saturation. Post-immersion, the specimens were carefully cleaned, and the resulting mass increase was measured after 28 days under saturated-surface-dry conditions.

After 90 days of curing, the specimens were exposed to 300 freeze–thaw cycles between −15 °C and +15 °C. Their strengths were re-evaluated post-exposure to assess residual mechanical properties and determine frost resistance.

### 2.4. Correlation Analysis

The correlation analysis examined the quantitative interrelationships between key properties to derive meaningful insights into the influence of OPA incorporation on the fresh and hardened performance of sustainable mortars. In particular, this study focused on the following:

- FT vs. WBD to assess how particle morphology and packing influence workability;
- Residual vs. 90-day compressive and flexural strengths to model freeze–thaw durability;
- CS28 vs. WA to explore the interdependence between mechanical performance and permeability.

## 3. Results and Discussion

Table 4 summarizes the experimental results of this study.

### 3.1. Wet Bulk Density

**Figure 3:**
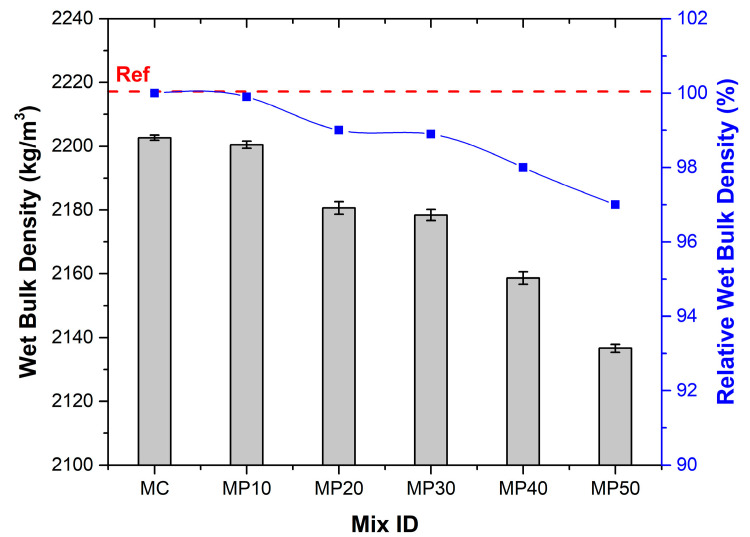
Relative wet bulk density results.

Figure 3 illustrates the decreasing trend in wet bulk density (WBD) as the replacement of OPC with OPA increases. The reference mixture MC recorded the highest wet bulk density (2202 kg/m^3^), which decreased by 3.0% to 2136 kg/m^3^ at 50% OPA replacement (MP50). This trend is attributed primarily to the lower specific gravity of OPA (2.18 g/cm^3^) compared to OPC (3.09 g/cm^3^). The OPC substitution with lighter OPA reduces the overall mass per unit volume. Moreover, the high porosity and irregular morphology of OPA particles hinder effective packing, introducing more voids and thus lowering bulk density. This trend aligns with OPA’s lower particle density (2.18) versus OPC (3.09) and its porous structure [19]. The irregular particle morphology [14,17] further hinders optimal packing, increasing void content at higher replacements.

### 3.2. Flow Time

**Figure 4:**
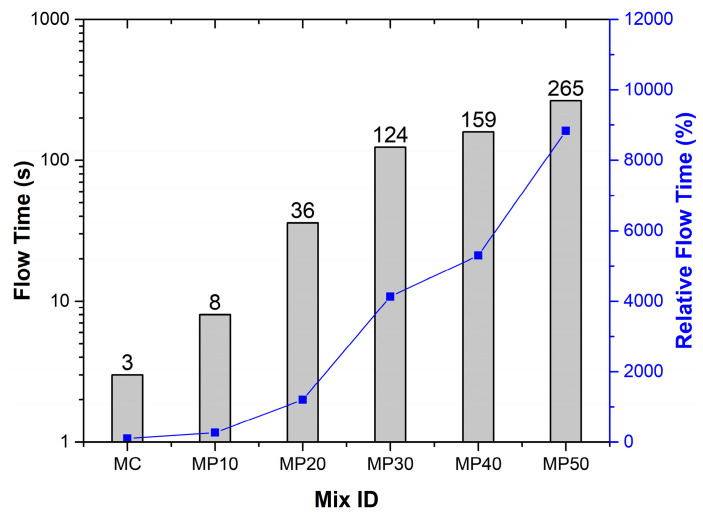
Flow test results.

The flow time results are presented in Figure 4. They indicate a steady increase with a higher OPA content from 3 s in MC to 265 s in MP50. This reflects a substantial reduction in workability.

The angular and porous nature of OPA particles, internal friction, and water demand limit the mortar’s ability to flow. At up to 10% OPA (MP10), the impact on flow was minimal, suggesting acceptable workability at lower substitution levels. Studies have shown that OPA particles have highly angular, irregular, and elongated shapes compared to the spherical particles of OPC [28]. Additionally, OPA has a wide particle size distribution, including coarser particles whose angularity, irregularity, and wider size range increase inter-particle friction within the mortar, hindering the flow and mobility of the mix and requiring more time to reach the same flow diameter. The irregular OPA particles also absorb more water. These effects collectively prolong FT with increasing OPA incorporation. These findings align with [14,28,29,30,31]. Nonetheless, up to 10% OPA replacement (MP10) showed only a marginal increase in FT compared to MC. While ASTM C1437 does not specify minimum or maximum flow time requirements, practical construction applications typically recommend flow times between 15 and 60 s for adequate workability and placement efficiency, indicating that OPA replacements beyond 20% may present workability challenges in field applications.

### 3.3. Compressive Strength

**Figure 5:**
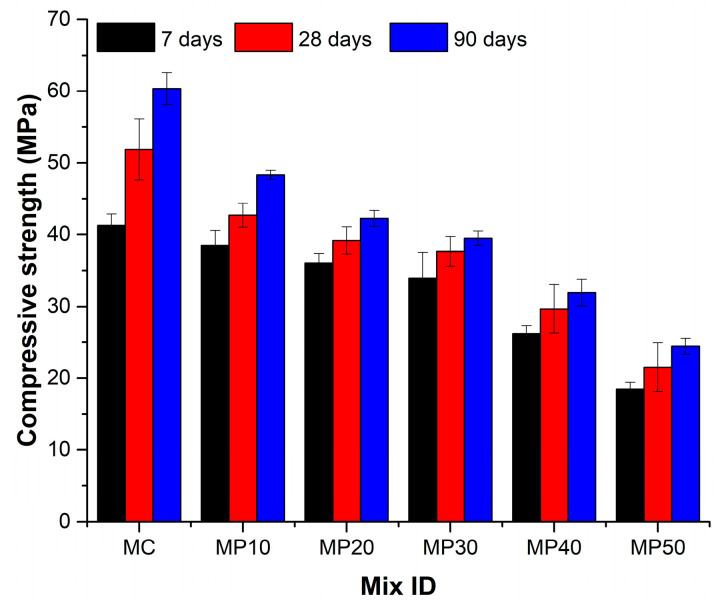
Compressive strength at 7, 28, and 90 days.

In Figure 5, the compressive strength declines with increasing OPA content across all curing ages. The control mix exhibited strengths of 41.27 MPa (7 days), 51.86 MPa (28 days), and 60.35 MPa (90 days). At 50% OPA (MP50), these values dropped to 18.47 MPa, 21.52 MPa, and 24.42 MPa, respectively—a reduction of over 55%.

The superior early and later age compressive performance of MC can be attributed to the hydration of abundant calcium silicate phases in OPC. Upon hydration, C2S and C3S form calcium silicate hydrates (C-S-H) that impart higher compressive strength [32].

In contrast, the 50% OPA mixture (MP50) exhibited significantly lower strengths of 18.47 MPa, 21.52 MPa, and 24.42 MPa at 7, 28, and 90 days, respectively. This drastic 55–60% strength reduction for MP50 is likely due to a reduced availability of reactive calcium silicates and slower pozzolanic reactions of OPA [29,33]. Additionally, the higher porosity and surface roughness of OPA particles may cause poor consolidation and increased flaws in the microstructure [34,35,36].

The marginal 7% early age strength reduction for MP10 compared to MC indicates that limited OPA incorporation up to 10% retains enough hydrating compounds from OPC for adequate early compressive strength development. The porous structure and irregular morphology of OPA particles likely have a negligible influence at such low replacement levels. Among the intermediate OPA content mixes, MP20 achieved reasonable strengths of 36.04 MPa, 39.18 MPa, and 42.26 MPa at 7, 28, and 90 days, respectively. The 12–16% strength reduction compared to MC can be attributed to an optimal balance between OPC hydration and OPA pozzolanic reaction at the 20% replacement level, without significant microstructural deficiencies.

Notably, strength continued to develop up to 90 days in all mixes, indicating sustained pozzolanic activity of OPA over time. MC registered a 32% strength increase between 7 and 90 days, while MP50 showed a 24.4% increase during the same period. The additional C-S-H gel formation fills pores and enhances the microstructure quality [28,37]. Thus, the increasing compressive strength trends indicate the pozzolanic behavior of OPA as a supplementary cementitious material.

EN 196-1 does not specify minimum compressive strength requirements as it focuses on test methodology; building codes typically require minimum 28-day compressive strengths of 20–25 MPa for structural mortar applications. All mixes achieved the minimum threshold of 21.52 MPa, confirming their suitability for construction use.

### 3.4. Flexural Strength

**Figure 6:**
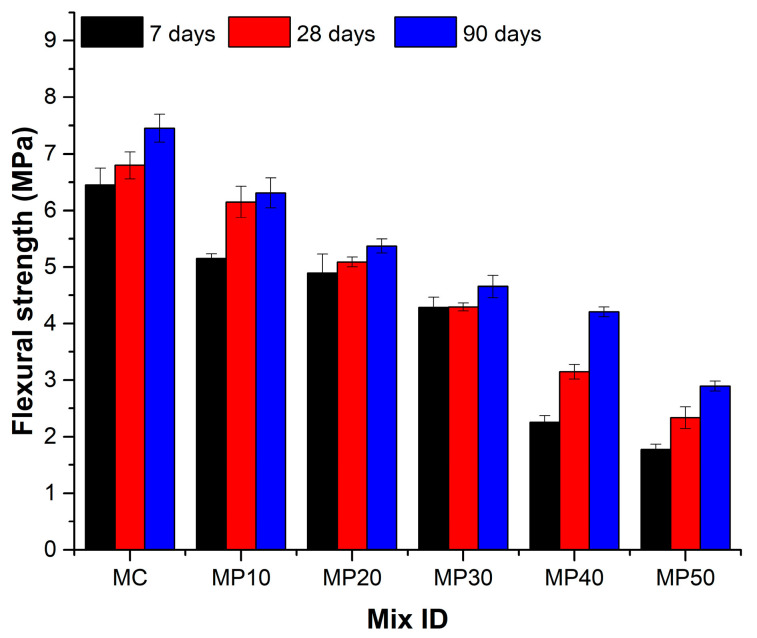
Flexural strength at 7, 28, and 90 days.

Figure 6 demonstrates that flexural strength followed a trend similar to compressive strength, declining as the OPA content increased.

The control mix achieved maximum strengths of 6.45 MPa (7 days), 6.80 MPa (28 days), and 7.45 MPa (90 days), while MP50 exhibited only 2.90 MPa at 90 days (FS90). This reduction is linked to decreased C–S–H gel formation and increased porosity [38]. In contrast, MP50 exhibited significantly lower strengths of 1.78 MPa, 2.33 MPa, and 2.90 MPa at 7, 28, and 90 days, respectively. The 72% decrease in early and 61% in later age strength is likely due to the slower pozzolanic reaction of OPA and the increased porosity of mixtures. The flexural strength reduction is attributable to a decrease in the bond between the OPA surface and the OPC paste [33]. Nevertheless, the results of MP10 reveal that limited OPA incorporation up to 10% does not compromise the flexural performance. The marginal 20% early age strength reduction for MP10 compared to MC indicates that low OPA ratios retain adequate hydration products for strength development. Among the intermediate mixes, MP40 exhibited a 120% enhancement in flexural strength from 2.26 MPa at 7 days to 4.21 MPa at 90 days due to the latent pozzolanic reactivity of OPA.

### 3.5. Water Absorption

**Figure 7:**
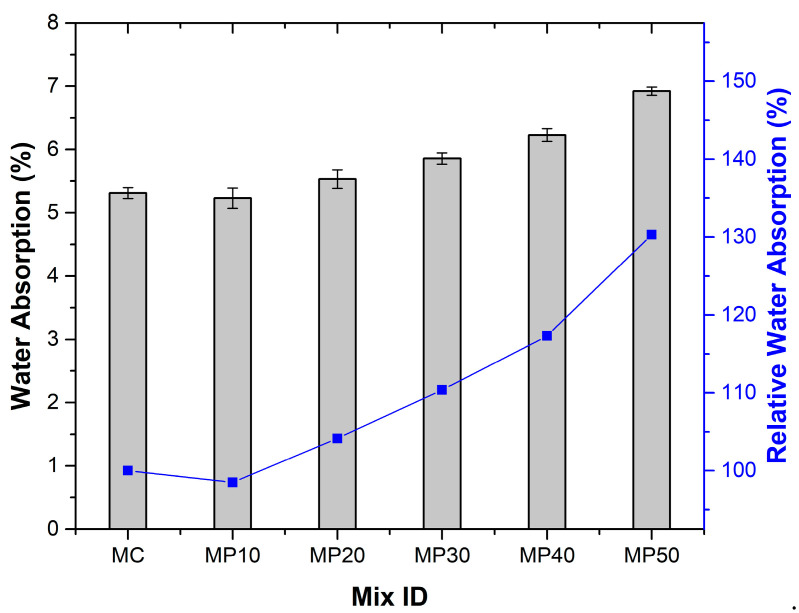
Water absorption results.

The 28-day WA results are in Figure 7, with relative water absorption values normalized to the control mix to enable direct comparison across different OPA replacement levels. MP10 showed slightly lower absorption (5.23%) than the control (5.31%), likely due to fine OPA particles filling voids and refining the pore structure. At high substitution levels, the increased porosity and weak bonding at the interfacial transition zone contributed to greater water ingress. Dahim et al. [39] similarly observed a parallel pattern.

As the OPA replacement level increased beyond 10%, WA rose (5.53% for MP20 and 5.86% for MP30) due to the weak adhesion between the highly porous and angular OPA particles and the cementitious matrix, leading to increased interfacial transition zone porosity [40]. Additionally, the higher water demand of OPA, due to its porosity, increased the effective w/c ratio, resulting in greater capillary porosity [19]. MP40 and MP50 recorded the highest absorption, reflecting the diminished microstructural compactness at elevated OPA dosages. The high angularity and surface roughness of OPA also increase pore interconnections [41].

EN 13057 establishes the test methodology for measuring water absorption but does not specify maximum allowable limits; however, durability guidelines typically recommend water absorption values below 10–15% for adequate long-term performance. All tested mixes achieved a maximum absorption of 6.92%, well within the acceptable ranges for durable mortar applications.

### 3.6. Freeze–Thaw Resistance

**Figure 8:**
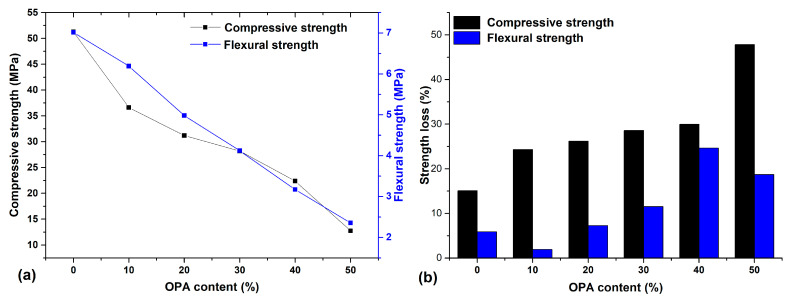
Freeze–thaw resistance results: (**a**) compressive and flexural strengths results; (**b**) strength loss.

Table 5 and Figure 8 present the 90-day residual mechanical strengths following 300 freeze–thaw cycles.

The control mix (MC) retained 85% of its compressive strength, while MP10 retained 76%. The MC performance can be attributed to the adequate formation of frost-resistant calcium silicate hydrates due to the hydration of reactive compounds in OPC [42]. Residual compressive strength (RCS) declined with increasing OPA, dropping to just 52% in MP50. This vulnerability is associated with higher porosity, poor interfacial bonding, and inadequate C–S–H formation. The drastic loss of strength with higher OPA proportions can be attributed to inadequate hydration products to resist frost damage [43]. The increased porosity and poor interfacial transition zone associated with a greater OPA content likely caused accelerated microcrack formation and propagation under freezing–thawing cycles [44].

Residual flexural strength (RFS) followed a similar pattern. MC showed minimal degradation (6%), and MP10 retained 98% of its pre-exposure strength. However, strength losses increased significantly at higher OPA levels, with MP50 experiencing a 19% drop. The results confirm that OPA contents beyond 20–30% impair the freeze–thaw durability.

The poor interfacial transition zones (ITZs) and microcrack development associated with the high porosity and angular morphology of OPA particles [45] contribute to weak interfaces and propagate cryogenic cracks [46].

Freeze–thaw resistance testing standards, such as ASTM C666 [47], typically require durability factors above 60–80% (equivalent to less than 20–40% strength loss) for acceptable frost resistance. The observed 48% strength loss indicates that higher OPA replacement levels may compromise freeze–thaw durability and should be limited for applications in freeze–thaw environments.

### 3.7. Interpretation of the Results

**Figure 9:**
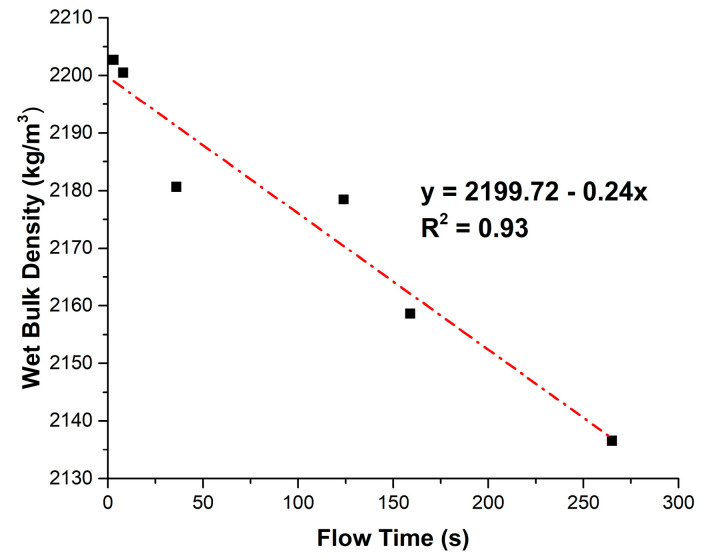
Relationship between wet bulk density and flow time.

Figure 9 shows the relationship between wet bulk density and flow time.

A linear regression curve with strong correlation (R^2^ = 0.93) was identified (Equation (1)) for WBD in kg/m^3^ and FT in s:

WBD = 2199.72 − 0.24 FT(1)

The highly angular, flaky, and elongated morphology of OPA particles increases inter-particle friction and reduces particle mobility within the fresh mortar matrix [48]. The irregular shapes tend to interlock, resisting flow. This effect is exacerbated by the rough surface texture of OPA particles, which further impedes sliding and rearrangement. Additionally, the porous structure of OPA particles enables a higher water demand than OPC particles [49]. The higher water demand of OPA to reach a homogeneous state contributes to increasing FT. Furthermore, the lower specific gravity of OPA compared to OPC also causes a reduction in WBD.

**Figure 10:**
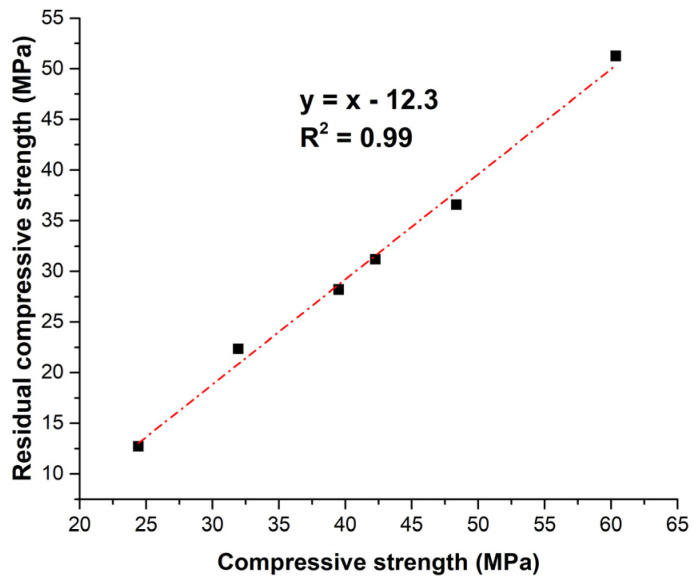
Relationship between compressive strength at 28 days and water absorption.

The correlation between CS28 and WA was established through regression analysis, as shown in Figure 10.

The most suitable model is exponential, according to Equation (2):

CS28 = 3873.88 WA^−2.66^ (2)

The square correlation coefficient R^2^ = 0.87 indicates a good fit of the findings with CS28 in MPa. The relationship demonstrates that small gains in compressive strength corresponded to disproportionately large reductions in WA. This supports the notion that moderate OPA incorporation can refine pore structure and reduce permeability, although excessive replacement increases porosity and weakens the matrix. Tailoring the OPA content to balance pozzolanic pore refinement and porosity addition is critical.

**Figure 11:**
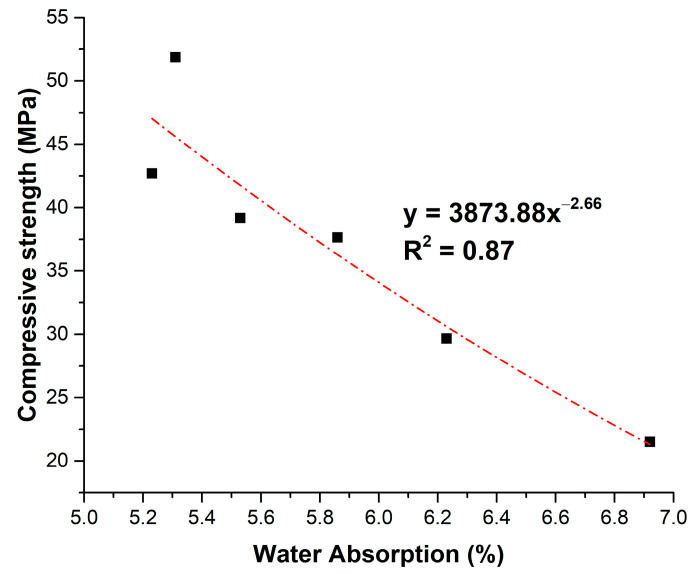
Relationship between residual compressive strength and compressive strength at 90 days.

Figure 11 displays the linear relationship between the residual compressive strength after 300 freeze–thaw cycles and the CS90 before exposure (Equation (3)).

RCS = CS90 − 12.3(3)

The near-perfect linear correlation (R^2^ = 0.99) between the variables in MPa suggests that residual compressive strength after freeze–thaw exposure is primarily governed by the initial 90-day strength. The empirical model implies a consistent strength loss regardless of mix composition.

**Figure 12:**
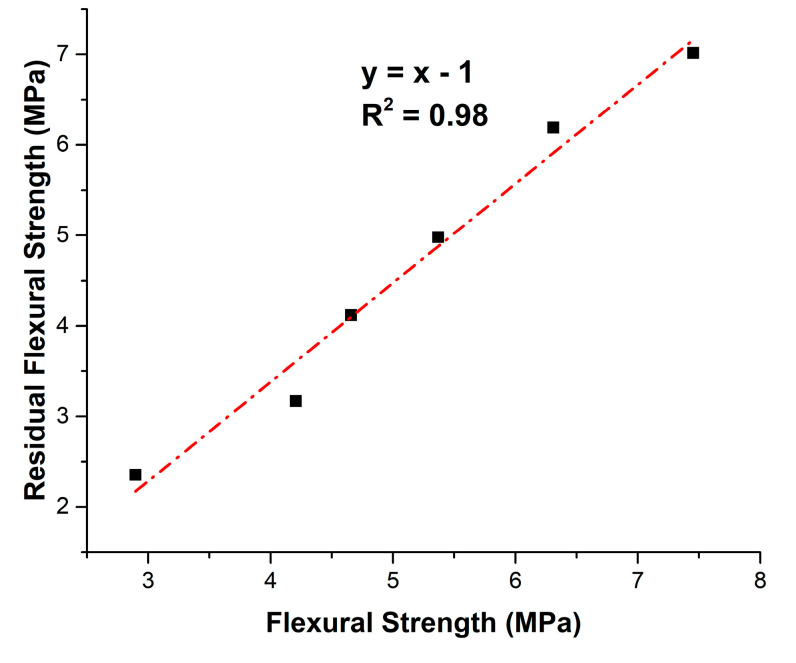
Relationship between residual flexural strength and flexural strength at 90 days.

Figure 12 illustrates the linear correlation (Equation (4)) between RFS after 300 freeze–thaw cycles and FS90.

RFS = FS90 − 1,(4)

A strong linear relationship (R^2^ close to 1) was found for the variables in MPa. The 1 MPa reduction indicates a proportional deterioration influenced by pre-exposure strength and inherent freeze–thaw damage mechanisms. Replacement levels up to 20% OPA maintained FS90 close to the control mix and supported better freeze–thaw resistance, while higher levels reduced strength and durability due to weakened hydration and increased porosity.

### 3.8. Environmental Analysis

Finally, a comprehensive techno-environmental assessment was carried out to integrate the dual objectives of mechanical performance and environmental sustainability. Olive pomace ash (OPA) demonstrated significant potential for cement manufacturing. Based on the mechanical performance and compositional criteria defined in EN 197-1 [50], the results support the feasibility of incorporating OPA into cement formulations. These findings suggest opportunities for further optimization and new cement types with OPA, contributing to more sustainable and resource-efficient binder systems. Cement production consumes about 800 and 1200 kWh/t and emits about 400 and 600 kg CO_2_/t [51,52,53,54]. On the other hand, the thermal treatment and grinding of olive pomace ash requires a lower thermal energy demand, estimated at 400 to 600 kWh/t [55]. In Algeria, where electricity generation is primarily based on natural gas, the greenhouse gas emissions associated with OPA production are 200–300 kg CO_2_/t. Table 6 lists the CO_2_ emissions and energy consumption of the tested mixtures.

The findings demonstrate that the partial replacement of Portland cement with olive pomace ash (OPA) results in a substantial decrease in CO_2_ emissions, highlighting its potential as a low-carbon alternative. As the substitution rate increases (from MP10 to MP50), a gradual reduction in energy consumption and CO_2_ emissions is observed. For example, at 10% substitution (MP10), the grinding energy is 953.5 kWh/t, resulting in 476.75 kg CO_2_/t, while at 50% substitution (MP50), the energy drops to 767.5 kWh/t, and the emissions are 383.75 kg CO_2_/t. These results highlight the environmental benefits of incorporating OPA in cement formulations, particularly in terms of reduced energy demand and a lower carbon footprint, thereby contributing to more sustainable construction materials. Although a reduction in compressive strength was observed across all mixtures, the mechanical performance remained acceptable. These results suggest that OPA can contribute to environmentally friendly cementitious materials with mechanical properties comparable to conventional systems.

## 4. Conclusions

Biomass ashes, such as olive pomace ash (OPA), present a promising solution by reducing cement use and diverting organic waste from landfilling through recycling and valorization. This study investigated the effects of partially replacing OPC with OPA in mortar formulations at varying replacement levels (0% to 50%). The key findings are as follows:

- Workability improved with OPA incorporation up to 20%, evidenced by a higher flow time and lower wet bulk density than the control mixture (36 s vs. 3 s, and 2180 kg/m^3^ vs. 2202 kg/m^3^). Beyond this level, workability significantly deteriorated.
- Mechanical properties, including compressive and flexural strength, declined with increasing OPA content (CS28 is 51.86 MPa in MC and 21.52 MPa in MP50). However, up to 20% of replacements retained sufficient strength for practical use, balancing environmental benefits and performance.
- Water absorption increased with OPA content, particularly above 30% (5.23% for MC and 5.86% for MP30). WA remained within tolerable limits for mixes containing up to 40% OPA.
- Durability under freeze–thaw conditions was maintained reasonably well for mixes with up to 30% OPA, though higher replacement levels led to notable strength losses after exposure. The control mix (MC) retained 85% of its compressive strength, while MP10 retained 76%. Regarding the residual flexural strength, MC showed minimal degradation (6%), and MP10 retained 98% of its pre-exposure strength.
- Correlation analyses provided insights into quantitative relationships between flow time and wet bulk density, compressive strength and water absorption, and residual and initial compressive and flexural strength.

Olive pomace ash can be successfully utilized as a sustainable SCM in controlled proportions to reduce the environmental footprint of cement-based materials and support circular economy strategies by recycling agricultural waste. Future research should focus on long-term durability assessments, physico-chemical and microstructural characterization of OPA and its interaction with cementitious matrices, and optimization of OPA processing to enhance performance and reactivity. Additionally, studies on large-scale applications and technical standards will be essential to support the broader adoption of OPA in sustainable construction.

## Figures and Tables

**Table 1:** Chemical composition of the OPC.

Oxides	SiO_2_	Al_2_O_3_	Fe_2_O_3_	CaO	MgO	SO_3_	K_2_O	Na_2_O	L.O.I.
%	19.4	4.74	3.25	63.18	3.2	2.56	0.63	0.25	2.67

**Table 2:** Chemical composition of the OPA.

Oxides	SiO_2_	Al_2_O_3_	Fe_2_O_3_	CaO	MgO	SO_3_	K_2_O	Na_2_O
%	11.5	2.82	1.48	10.1	1.49	3.27	0.331	2.14

**Table 3:** Formulation details of mortar mixtures.

Mix ID	OPC (g)	OPA (%)	OPA (g)	Water (g)	W/B	Sand (g)
MC	450	0	0	225	0.50	1350
MP10	405	10	45			
MP20	360	20	90			
MP30	315	30	135			
MP40	270	40	180			
MP50	225	50	225			

**Table 4:** Experimental results.

Mix ID	WBD (kg/m^3^)	FT (s)	CS7 (MPa)	CS28 (MPa)	CS90 (MPa)	FS7 (MPa)	CF28 (MPa)	CF90 (MPa)	WA (%)
MC	2202	3	41.27	51.86	60.35	6.45	6.8	7.45	5.31
MP10	2200	8	38.48	42.7	48.36	5.15	6.15	6.31	5.23
MP20	2180	36	36.04	39.18	42.25	4.9	5.09	5.37	5.53
MP30	2178	124	33.96	37.66	39.49	4.29	4.293	4.66	5.86
MP40	2158	159	26.17	29.67	31.93	2.25	3.149	4.21	6.23
MP50	2136	265	18.47	21.52	24.42	1.77	2.33	2.89	6.92

**Table 5:** Residual strengths and strength losses results.

Mix ID	Compressive Strength		Flexural Strength	
	Residual (MPa)	Loss (%)	Residual (MPa)	Loss (%)
MC	51.27	15.05	7.02	5.86
MP10	36.6	24.33	6.19	1.92
MP20	31.2	26.17	4.98	7.26
MP30	28.2	28.59	4.12	11.53
MP40	22.36	29.98	3.17	24.65
MP50	12.74	47.84	2.35	18.7

**Table 6:** Environmental performances.

Mix ID	Energy Consumption (kWh/t)	Emission (kg CO_2_/t)
MC	1000	500
MP10	953.5	476.75
MP20	907	453.5
MP30	860.5	430.25
MP40	814	407
MP50	767.5	383.75

## Data Availability

The original contributions presented in the study are included in the article, further inquiries can be directed to the corresponding author.

## Abbreviations

The following abbreviations are used in this manuscript:

OBA	olive biomass ash
OPA	olive pomace ash
OPC	Ordinary Portland Cement
OWA	olive waste ash
SCM	supplementary cementitious material
CS28	28-day compressive strength
RCS	residual compressive strength
FS	flexural strength
CS90	90-day compressive strength
RFS	residual flexural strength
WA	water absorption
WBD	wet bulk density
